# Supplementary material for: Complexome profiling of the Chlamydomonas psb28 mutant reveals TEF5 as an early PSII assembly factor
Source: Plant Cell. 2025 Mar 17;37(6):koaf055. doi: 10.1093/plcell/koaf055 (PMC12164590; doi:10.1093/plcell/koaf055)
Supplement: koaf055_Supplementary_Data [file koaf055_supplementary_data.zip › psb28_interactive.zip!/plots/intensity/qualityPlots/peptides/OPR52_Cre12.g505150.t1.1.html]

### Cre12.g505150.t1.1

Trivial name: OPR52  
SeqCoverage [median]: 0.50  
Mass: 147.50 kDa  
  
 

| Sequence | MissedCleavage | Mass [Da] | wtIntensity | muIntensity | Score |
| --- | --- | --- | --- | --- | --- |
| LGEVAQR | 0 | 771.42 | 91051.59 | 113021.91 | 208.74 |
|  |  |  | 91051.6 | 113021.9 |  |
